# Integrative Analysis of Transcriptomics and Metabolomics Provides Insights into Meat Quality Differences in Hu Sheep with Different Carcass Performance

**DOI:** 10.3390/foods14142477

**Published:** 2025-07-15

**Authors:** Xiaoxue Zhang, Liming Zhao, Huibin Tian, Zongwu Ma, Qi Zhang, Mengru Pu, Peiliang Cao, Deyin Zhang, Yukun Zhang, Yuan Zhao, Jiangbo Cheng, Quanzhong Xu, Dan Xu, Xiaobin Yang, Xiaolong Li, Weiwei Wu, Fadi Li, Weimin Wang

**Affiliations:** 1 College of Animal Science and Technology, Gansu Agricultural University, Lanzhou 730070, China; zhangxx@gsau.edu.cn (X.Z.); c3264887984@outlook.com (P.C.); 2 State Key Laboratory of Herbage Improvement and Grassland Agro-Ecosystems, Key Laboratory of Grassland Livestock Industry Innovation, Ministry of Agriculture and Rural Affairs, Engineering Research Center of Grassland Industry, Ministry of Education, College of Pastoral Agriculture Science and Technology, Lanzhou University, Lanzhou 730020, China; zlmfxy1807285865@163.com (L.Z.); tianhb@lzu.edu.cn (H.T.); gsaumzw980406@163.com (Z.M.); 13040547928@163.com (Q.Z.); pumr2023@lzu.edu.cn (M.P.); zdy1213@163.com (D.Z.); 120220900701@lzu.edu.cn (Y.Z.); zhaoyuan_10@163.com (Y.Z.); 15117098920@163.com (J.C.); 18147121406@163.com (Q.X.); 15045093462@163.com (D.X.); yangxb0902@163.com (X.Y.); lixllil@163.com (X.L.); lifd@lzu.edu.cn (F.L.); 3 Institute of Animal Science, Xinjiang Academy of Animal Sciences, Urumqi 830011, China; wuweiweigp@foxmail.com

**Keywords:** Hu sheep, meat quality traits, carcass performance, transcriptomics, metabolomics

## Abstract

Meat quality is a critical determinant of consumer preference and economic value in the livestock industry. However, the relationship between carcass performance and meat quality remains poorly understood. In our study, we conducted an integrative analysis of transcriptomics and metabolomics to investigate the molecular mechanisms underlying meat quality differences in Hu sheep with high (HHS, *n* = 10) and low (LHS, *n* = 10) carcass performance. Phenotypic analysis revealed that the HHS group exhibited superior meat quality traits, including higher intramuscular fat (IMF) content (reflected in elevated marbling scores), along with lower shear force, drip loss, and cooking loss, compared to the LHS group. Transcriptomic analysis identified 376 differentially expressed genes (DEGs) enriched in pathways linked to lipid metabolism, such as the PPAR signaling pathway and long-chain fatty acid metabolic process. Weighted gene co-expression network analysis (WGCNA) revealed important modules and key genes (e.g., *ELOVL6*, *PLIN1*, and *ARHGEF2*) associated with meat quality traits. Metabolomic profiling identified 132 differentially accumulated metabolites (DAMs), with significant enrichment in amino acid metabolism pathways, including D-amino acid metabolism, arginine biosynthesis, and glycine, serine, and threonine metabolism. Integrative analysis of transcriptomic and metabolomic data highlighted six co-enriched pathways, such as the mTOR signaling pathway and amino acid metabolism, underscoring their role in regulating meat quality. These findings provide valuable insights into the genetic and metabolic networks driving meat quality variation and offer potential biomarkers for genetic selection and nutritional strategies to enhance both carcass yield and eating quality in Hu sheep. This research enhances knowledge of the molecular basis of meat quality and supports precision breeding in livestock production.

## 1. Introduction

Meat quality is a critical determinant of consumer preference and economic value in the livestock industry [1], particularly for sheep meat production. Key attributes such as tenderness, IMF content, marbling, and water-holding capacity are influenced by complex interactions between genetic factors, metabolic pathways, and postmortem biochemical processes [2]. While carcass-performance traits (e.g., carcass weight, length, dressing percentage, etc.) are often prioritized in breeding programs, their relationship with meat quality remains poorly understood. Thus, understanding the interrelationships between meat quality and carcass traits is essential for creating breeding strategies that enhance productivity while maintaining or improving both carcass and meat quality [3,4].

Hu sheep, an indigenous breed in China, are renowned for their excellent adaptability to the local environment, remarkable growth rates, and high reproductive efficiency. However, despite their economic importance, the relationship between carcass performance and key meat quality attributes within this breed remains poorly characterized. This knowledge gap hinders targeted breeding for both yield and eating quality. Therefore, this study was conducted specifically on Hu sheep to elucidate the molecular mechanisms underlying meat quality variation in relation to carcass performance. High-throughput omics approaches have significantly advanced the investigation of biological mechanisms related to economically significant traits [5,6,7]. Transcriptomics provides insights into gene expression patterns regulating muscle growth and development [8], while metabolomics captures dynamic changes in small molecules that directly reflect phenotypic outcomes [9,10]. Integrative analysis of these datasets has proven powerful in uncovering molecular networks driving meat quality in livestock. For instance, Yu et al. [5] conducted a comparative analysis of the transcriptome and metabolome between local and high-IMF cattle breeds, providing valuable insights for improving native cattle breeding and meat quality. Dan et al. [11] performed integrated transcriptomic and metabolomic analyses on Neijiang and Neijiang × Large White pigs, providing valuable information on the genetic and metabolic factors influencing meat quality and carcass traits. Previous studies in sheep have identified several genes linked to meat quality traits, including *PHGDH* [12], *OLFML3*, *ANGPTL2* [13], *PPARGC1A*, *WFIKKN2* [14], *FABP4*, *PLIN1*, and *PCK1* [15]. However, the key genes responsible for the differences in meat quality among sheep with varying carcass performance have yet to be identified, and their underlying genetic mechanisms need further investigation.

In this study, we identified DEGs and DAMs related to the meat quality traits of Hu sheep with different carcass performance, using transcriptomic and metabolomic analyses, respectively. By combining transcriptomic and metabolomic approach, we aimed to (1) identify key DEGs and DAMs between high- and low-carcass-performance sheep, (2) characterize co-expression networks linking hub genes to meat quality traits, and (3) reveal integrated pathways connecting transcriptional regulation with metabolic reprogramming. Our findings provide a systems-level understanding of the molecular mechanisms underlying meat quality variation, offering valuable biomarkers for genetic selection and nutritional strategies to optimize both carcass yield and eating quality in Hu sheep.

## 2. Materials and Methods

### 2.1. Experimental Animals, Tissue Collection, Phenotyping

Ninety-three Hu sheep were raised on Minqin Defu Agriculture Co., Ltd. (Wuwei, China). From 56 days old at weaning to 180 days old at slaughter, all lambs were raised under consistent conditions. They underwent 14 days of acclimatization, followed by 10 days of pre-testing and 100 days of experimentation. After slaughter through carotid artery exsanguination, the carcass weight, length, and chest circumference of all sheep were measured and recorded using a calibrated electronic scale and a soft measuring tape. Based on slaughter traits, the sheep were categorized into two groups: HHS (high-slaughter-performance Hu sheep, *n* = 10, with 5 males and 5 females; carcass weight = 28.06 ± 0.31 kg) and LHS (low-slaughter-performance Hu sheep, *n* = 10, with 5 males and 5 females; carcass weight = 19.96 ± 0.11 kg). The sheep were fasted for 12 h before slaughter, during which they had free access to water. After slaughter, the longissimus thoracis (LT) tissues of each animal was immediately collected and weighed. A portion was stored at −80 °C for transcriptome sequencing and qPCR, while the remaining part was used for meat quality analysis. Growth traits (including body weight, length, height, and chest circumference) and slaughter traits (including live weight before slaughter, backfat thickness, and GR value) were measured and recorded using calibrated electronic scales and rulers. Among them, the backfat thickness was determined at the junction of the 12th and 13th ribs using electronic calipers. The girth rib (GR) value, taken 11 cm from the midpoint over the 12th rib, was used to represent carcass fat content and was directly assessed on all carcasses [16,17]. A *t*-test was used to compare the phenotypic traits between the HHS and LHS groups.

### 2.2. Meat Quality Measurements

Drip loss was measured following Honikel’s method [18] with slight modifications. After slaughter, the LT tissue was accurately weighed and suspended in plastic bottles, then stored at 4 °C for 24 h to measure drip loss. The drip loss percentage was determined through the formula (Δm/m_0_) × 100, where Δm represents the mass difference before and after the dripping process, and m_0_ denotes the initial weight. To determine cooking loss, three meat samples, each weighing about 30 g (weighed using an analytical balance) and of identical size, were randomly taken from the LT tissue of each sheep. The samples were sealed in self-sealing bags and immersed in a preheated water bath set to 80 °C, where they were heated for 45 min. After heating, the samples were removed and hung in a cool, dry place to air dry until reaching room temperature, and the weight of the cooked samples was then recorded. All experimental and parallel samples were processed in a single batch. Cooking losses were determined by measuring the weight difference before and after cooking as a percentage. Six random samples (with each sample weighing about 50 g) were taken from the meat samples using a circular core sampler with a diameter of 1.27 cm, parallel to the muscle fibers. The shear force was measured using a Warner–Bratzler shear machine (GR Manufacturing, Manhattan, KS, USA). Marbling was rated on a scale from 0 to 5, with 5 representing the highest level of marbling. The intramuscular fat and moisture contents in the LT were determined using a FoodScan Meat Analyzer (FOSS Analytical A/S, Hillerød, Denmark).

### 2.3. Measurement of Muscle Fiber Indicators

Following slaughter, muscle tissues from all sheep were collected and fixed overnight in 10% neutral formaldehyde. Fixed tissues underwent ethanol dehydration and paraffin embedding. Serial transverse sections were then prepared. Muscle fiber indicators (including muscle fiber diameter, area, and perimeter) were assessed using a digital trinocular microscope (BA210Digital, MacAudi Industrial Co., Ltd., Shanghai, China), with images captured and analyzed using Motic Images Advanced 3.2 software.

### 2.4. Library Preparation and RNA-Seq

From the longissimus thoracis of 20 animals, RNA was purified with TransZol (TransGen Biotech, Beijing, China), following the standard procedure. Using the RNA Nano 6000 Assay Kit, RNA quality was analyzed on the Agilent Bioanalyzer 2100 system. The RNA was maintained at −80 °C until further analysis. cDNA libraries were constructed with the NEBNext^®^ UltraTM RNA Library Prep Kit for Illumina^®^ (NEB, San Diego, CA, USA), in accordance with the manufacturer’s instructions. mRNA was first isolated from total RNA using poly-T oligo-attached magnetic beads. The first-strand cDNA was synthesized using random hexamer and M-MuLV Reverse Transcriptase. The second-strand cDNA was generated using DNA Polymerase I and RNase H. To preferentially obtain cDNA fragments ranging from 250 to 300 bp, the library underwent purification using the AMPure XP system. Finally, the resulting libraries were processed using the Illumina Novaseq Xplus platform, generating 150 bp paired-end sequences.

### 2.5. RNA-Seq Data Analysis

Raw sequencing reads were evaluated using FastQC (v0.12.1, https://www.bioinformatics.babraham.ac.uk/projects/fastqc/ (accessed on 26 February 2025)). Subsequently, the raw reads were processed using fastp (v0.22.0) [19] to remove adapters, poly-N sequences, and low-quality reads, resulting in clean data. Only high-quality clean reads were retained for analysis and then aligned to the ovine reference genome Oar_rambouillet_v1.0 (GCA_002742125.1, https://www.ncbi.nlm.nih.gov/ (accessed on 16 March 2025)) using Hisat2 (v2.2.1) software. Subsequently, we normalized the raw gene counts with the transcripts per million (TPM) method, employing an in-house script. Principal component analysis (PCA) was performed on the all genes using vegan R package (v2.6.10). All genes were transformed using log2(TPM + 1).

Transcriptomic profiling of HHS and LHS groups was conducted employing the DESeq2 package (v1.44.0) within the R software (v4.4.1) environment. After filtering out low-expressed genes (with counts < 10 in more than 20% of the samples), a total of 15,529 gene profiles were retained for subsequent analysis. We identified significantly differentially expressed genes (DEGs) between two groups with thresholds of an *p*-value < 0.05 and |fold changes| > 1.5. The enrichment analysis of all genes was performed using gene ontology (GO) and Kyoto Encyclopedia of Genes and Genomes (KEGG) with the clusterProfiler R package (v4.6.0) [20]. The threshold for statistical significance in the enrichment analysis was set at an adjusted *p*-value of less than 0.05.

### 2.6. Weighted Correlation Network Analysis

We conducted weighted gene co-expression network analysis (WGCNA) using the WGCNA (v1.73) [21] based on the aggregated expression matrix from muscle tissue. Transcriptomic features exhibiting a cumulative read count exceeding 10 across all samples and demonstrating a median absolute deviation (MAD) above the 25th percentile (MAD > 0.01) were retained for subsequent analysis, yielding 17,383 genes for the weighted gene co-expression network analysis. The ideal soft threshold power (β = 1–30) was chosen according to the scale-free distribution. A signed adjacency matrix was computed based on pairwise gene expression correlations and transformed into a topological overlap matrix (TOM), which was used to perform hierarchical clustering and identify gene modules. The total of sixteen traits (including four growth traits, five slaughter traits, and seven meat quality traits) were used as trait files to evaluate module–trait relationships. Significant associations between modules and traits were defined as *p*-value < 0.05, where * denotes *p*-value < 0.05, and ** denotes *p*-value < 0.01. Cytoscape (v3.10.0) was employed to visualize the regulatory network of co-expressed genes.

### 2.7. Metabolite Extraction for LC-MS/MS Analysis

All longissimus thoracis samples used in RNA-Seq were also employed for metabolomics analysis. The metabolite extraction procedure followed is outlined as follows: (1) 30 mg of the sample were placed into a 2 mL centrifuge tube, and 200 µL of pre-chilled water was added. The mixture was then ground using a tissue grinder for 60 s, and the process was repeated once; (2) 800 µL of an extraction solution with a 1:1 methanol/acetonitrile ratio and the internal standard was added to the sample; (3) the sample was subjected to ultrasound treatment for 30 min, followed by freezing at −20 °C for 30 min; (4) the sample was centrifuged at 12,000 rpm for 10 min at 4 °C; (5) 800 µL of the supernatant was carefully collected, and the extract was vacuum-concentrated to dryness; (6) 150 µL of 50% methanol (containing 5 ppm 2-chlorophenylalanine) was added to reconstitute the sample, followed by vortexing for 30 s; (7) following centrifugation at 12,000 rpm and 4 °C for 10 min, the resulting supernatant was membrane-filtered; (8) the filtrate was transferred into an injection vial, and 10–20 µL from every sample was combined to create a quality control (QC) sample. The QC sample, along with the other samples, was then analyzed using LC-MS/MS.

### 2.8. Statistical Analysis of the Metabolites

The XCMS software (v4.3) [22] was employed to process the raw data of both positive and negative ion metabolite concentrations, encompassing peak alignment, retention time calibration, and peak area determination. Multivariate analyses encompassing PCA, partial least squares–discriminate analysis (PLS-DA), and orthogonal projections to latent structures–discriminant analysis (OPLS-DA) were executed utilizing the R environment. The first principal component of the variable importance in projection (VIP) was derived from the OPLS-DA to enhance the analysis. Differentially accumulated metabolites (DAMs) were identified using the following criteria: VIP  >  1 and *p*-value  <  0.05. A volcano plot and a hierarchical cluster heatmap were constructed to illustrate the results. The differentially expressed metabolic pathways were analyzed using the KEGG database [23]. Statistical significance in the enrichment analysis was determined with an adjusted *p*-value of less than 0.05.

### 2.9. Joint Analysis of the Transcriptomic and Metabolomic Data

To gain a deeper understanding of the transcriptional regulation mechanisms within the metabolic pathways, we combined transcriptome and metabolic data. Firstly, we calculated Pearson correlations between all DEGs and DAMs based on gene expression levels and the relative abundances of metabolites using the corr.test function from the psych package (v2.5.3) in the R environment (v4.4.1). Significant correlations were defined at two thresholds: *p*-value < 0.05 (marked with *) and *p*-value < 0.01 (marked with **). We present the significant gene-metabolite pairs related to meat quality. Cytoscape (v3.10.0) was used for the visualization of the association network diagram. Subsequently, we performed a joint analysis of the differential metabolites and hub genes identified through WGCNA, highlighting their shared pathways.

### 2.10. Validation of the DEGs Identified from RNA-Seq Data

RNA was extracted from longissimus thoracis tissue using TransZol (TransGen Biotech, Beijing, China) according to the manufacturer’s protocol. Briefly, tissue samples were homogenized in 1 mL of TransZol reagent using a sterile pestle. After incubating at room temperature for 5 min, 0.2 mL of chloroform was added, and the mixture was shaken for 15 s, incubated for 3 min, and then centrifuged at 12,000× *g* for 15 min at 4 °C. The aqueous phase was transferred to a new RNase-free tube, and RNA was precipitated by adding an equal volume of isopropanol and incubating for 10 min at room temperature. The RNA pellet was collected by centrifugation at 12,000× *g* for 10 min at 4 °C, washed with 1 mL of 75% ethanol, and air-dried. Finally, the RNA pellet was dissolved in 30 μL of RNase-free water. Subsequently, cDNA was synthesized from total RNA using a reverse transcription kit (Takara, Dalian, China) following the manufacturer’s protocol. Quantitative PCR analysis was performed using a SYBR premix Ex Taq™ kit (Takara Biotechnology) on the LightCycler 480 II thermal cycler (Roche, Mannheim, Germany). The thermal cycling conditions comprised an initial denaturation at 95 °C for 3 min, followed by 40 cycles of 95 °C for 15 s, annealing at optimized temperatures for 15 s, and extension at 72 °C for 20 s. Primer sequences were designed using bioinformatics software (Oligo 7.0 and Primer 5) with stringent parameters to ensure specificity (Table 1). The *UXT* gene of sheep was selected as the endogenous control based on its stable expression across experimental conditions. Each sample was analyzed in quadruplicate to ensure technical reproducibility. Relative expression levels of genes were quantified through the 2^−ΔΔCT^ approach [24].

## 3. Results

### 3.1. Correlation Analysis Between Muscle Fiber Indicators and Growth, Carcass, and Meat Quality Traits

**Figure 1:**
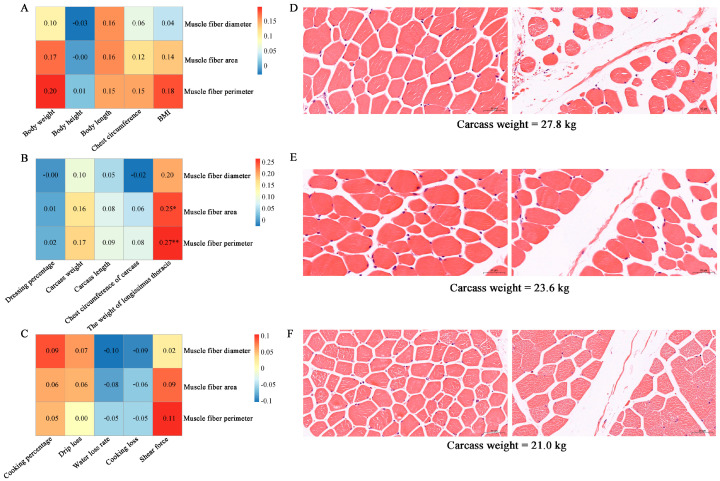
Correlation analysis of muscle fiber characteristics with growth, carcass, and meat quality traits. (**A**) Correlation analysis between muscle fiber indicators and growth traits. (**B**) Correlation analysis between muscle fiber indicators and carcass traits; * indicates adjusted *p*-value < 0.05; ** indicates adjusted *p*-value < 0.01. (**C**) Correlation analysis between muscle fiber indicators and meat quality traits. (**D**–**F**) Muscle fiber characteristics of sheep with different carcass performance (carcass weights are 27.8, 23.6, and 21.0 kg, respectively).

Muscle fiber characteristics are crucial factors influencing both meat quality and yield [25]. We performed Pearson correlation analysis using the psych [26] package (v2.5.3) in R (v4.4.1) environment to investigate the relationships between muscle fiber indicators and growth, carcass, and meat quality traits in 93 Hu sheep. A adjusted *p*-value < 0.05 was considered statistically significant for correlations. Regarding growth traits, muscle fiber diameter, area, and perimeter showed weak positive correlation trends with body weight, body length, chest circumference, and BMI (body mass index, Figure 1A), though these associations were not statistically significant (*p*-value > 0.05). In terms of carcass performance, muscle fiber indicators showed a significant positive correlation (*p*-value < 0.05) with longissimus thoracis weight, while muscle fiber diameter exhibited weak negative correlation trends with dressing percentage and carcass chest circumference that did not reach statistical significance (*p*-value > 0.05, Figure 1B). Regarding meat quality traits, muscle fiber indicators displayed non-significant weak positive correlation trends with cooking percentage, drip loss, and shear force and non-significant weak negative correlation trends with water loss rate and cooking loss (all *p*-value > 0.05, Figure 1C). Figure 1D–F show the muscle fiber characteristics of Hu sheep with different carcass weights.

### 3.2. Comparison of Meat Quality Characteristics in Sheep with Different Carcass Performance

**Figure 2:**
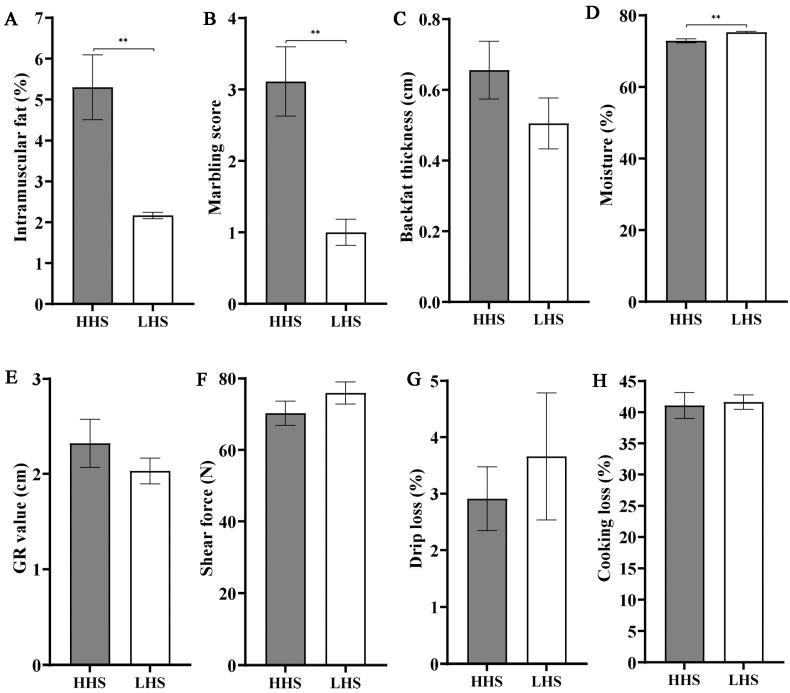
Phenotypic characteristics comparison between HHS and LHS groups. (**A**) Intramuscular fat content; (**B**) marbling score; (**C**) backfat thickness; (**D**) moisture content; (**E**) GR value; (**F**) shear force; (**G**) drip loss; (**H**) cooking loss. Data expressed as mean ± SEM. ** indicates *p*-value < 0.01.

As shown in Table 2, the growth traits (including body weight, length, height, chest circumference, and body mass index) and slaughter traits (including live weight before slaughter, carcass weight, carcass length, and chest circumference of carcass) in the HHS group were significantly higher than those in the LHS group (*p*-value < 0.05). For meat quality traits, the fat content and marbling score in the HHS group were considerably greater compared to the LHS group (*p*-value < 0.05, Figure 2A,B). In contrast, the moisture content in the LHS group exceeded that of the HHS group significantly (*p*-value < 0.05, Figure 2D). Additionally, the backfat thickness and GR value in the HHS group were greater than those in the LHS group, while the shear force, drip loss, and cooking loss were lower in the HHS group; however, the differences were not significant (*p*-value > 0.05, Figure 2C,E–H).

### 3.3. DEGs Identification and Transcriptome Analysis

**Figure 3:**
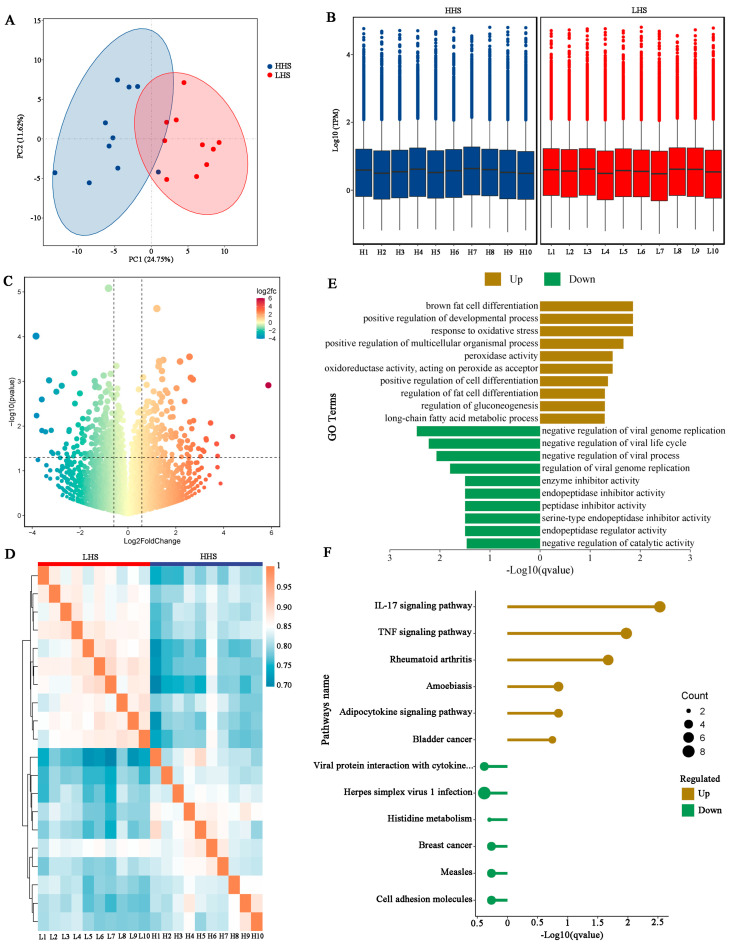
Transcriptome analysis of HHS vs. LHS comparison. (**A**) PCA clustering based on gene expression profiles. (**B**) Expression distribution boxplot using standardized TPM values. (**C**) Volcano plot of DEGs in the HHS vs. LHS comparison. (**D**) Pearson correlation heatmap of inter-sample expression patterns. (**E**) GO enrichment analysis of up-regulated and down-regulated DEGs. (**F**) KEGG pathway enrichment analysis of up-regulated and down-regulated DEGs.

PCA of normalized expression matrix showed a clear separation between the HHS and LHS groups (Figure 3A). To assess the consistency of gene expression distribution across biological replicates, a boxplot analysis was performed using standardized TPM values (Figure 3B). All samples exhibited similar median expression levels and interquartile ranges after normalization, confirming the comparability of expression data between groups. To compare the difference in muscle mRNA between the HHS and LHS groups, the thresholds of *p*-value < 0.05 and FC > 1.5 were used to identify the differentially expressed genes. In this study, we identified a total of 376 DEGs in the HHS vs. LHS comparison, including 188 up-regulated genes and 188 down-regulated genes in the HHS group (Figure 3C; Table S1). The correlation heatmap clearly shows a marked intergroup difference and strong intragroup similarity (Figure 3D). The up-regulated DEGs were primarily associated with the terms “brown fat cell differentiation”, “positive regulation of developmental process”, “response to oxidative stress”, “peroxidase activity”, and “positive regulation of cell differentiation”, while down-regulated DEGs were primarily enriched in “negative regulation of viral process”, “enzyme inhibitor activity”, “peptidase inhibitor activity”, and “negative regulation of catalytic activity” (Figure 3E; Table S2). The KEGG analysis indicated that the up-regulated DEGs were involved in the “IL-17 signaling pathway”, “TNF signaling pathway”, and “rheumatoid arthritis” pathways, while the down-regulated DEGs showed no significant enrichment in any pathway (Figure 3F; Table S3). Several important DEGs associated with these fat synthesis terms and pathways have been identified, such as *ADIPOQ*, *CEBPA*, *LEP*, and *PTGS2*, which have been reported to be related to intramuscular fat content [27], fat accumulation [17], and tail fat deposition [28].

### 3.4. Identification of Hub Genes Associated with Meat Quality Traits

**Figure 4:**
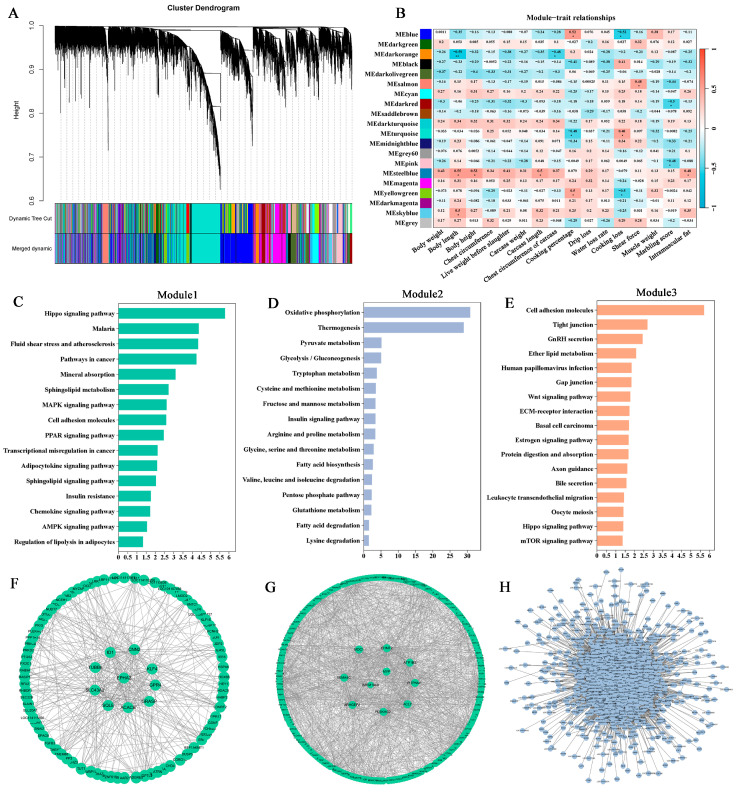
Weighted gene co-expression network analysis of genes. (**A**) Module clustering dendrogram with color-coded gene groups. (**B**) Relationship between the module eigengene and phenotypic traits; * indicates *p*-value < 0.05; ** indicates *p*-value < 0.01. (**C**–**E**) KEGG enrichment analysis of genes in the MEsteelblue/MEskyblue, MEblue/MEyellowgreen, and MEsalmon modules, respectively. (**F**–**H**) Interaction network of hub genes in the MEsteelblue, MEskyblue, and MEblue modules, with hub genes displayed individually. A greater number of lines between two genes indicates a higher potential for interactions.

Based on WGCNA analysis, we identified some key gene clusters related to sheep economic traits (Figure 4A). The optimal soft threshold was determined to be 8, as indicated in Figure S1A. Figure S1B,C also present the cluster dendrogram and network heatmap illustrating the relationships between modules. The MEsteelblue and MEskyblue modules were significantly correlated with growth traits in sheep. For meat quality traits, the MEblue and MEyellowgreen modules showed a significant association with cooking percentage and cooking loss, while the MEsalmon module was significantly correlated with shear force in sheep (Figure 4B). Subsequently, we performed KEGG enrichment analysis on these key modules. For MEsteelblue and MEskyblue modules, the genes are mainly enriched in the “mineral absorption”, “sphingolipid metabolism”, “MAPK signaling pathway”, “PPAR signaling pathway”, “adipocytokine signaling pathway”, and “regulation of lipolysis in adipocytes” pathways (Figure 4C). For MEblue and MEyellowgreen modules, the genes are mainly enriched in the “oxidative phosphorylation”, “pyruvate metabolism”, “tryptophan metabolism”, “cysteine and methionine metabolism”, “arginine and proline metabolism”, “pentose phosphate pathway”, and “fatty acid degradation” pathways (Figure 4D). For MEsalmon module, the genes are mainly enriched in the “cell adhesion molecules”, “tight junction”, and “gap junction” pathways (Figure 4E). To identify high-degree genes that play a critical role in the interaction network, network interaction data generated from WGCNA analysis were visualized using Cytoscape software. Subsequent analysis of the gene interaction network revealed that several key genes, such as *EPHA2* (degree = 41), *SLC43A2* (degree = 33), *CNN2* (degree = 44), *KLF4* (degree = 37), *GPR4* (degree = 17), *GRASP* (degree = 17), *ACACB* (degree = 30), *SEMA4C* (degree = 69), *ARHGEF2* (degree = 89), *MDC1* (degree = 64), *EHMT2* (degree = 107), and *ATP1B2* (degree = 90), were positioned at the center of the network (Figure 4F–H).

### 3.5. DEMs and Metabolome Analysis

**Figure 5:**
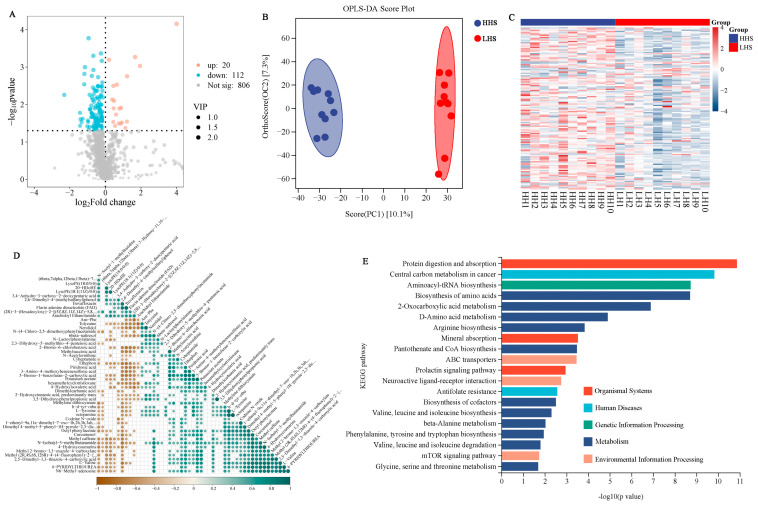
Metabolomic profiling and functional analysis of HHS vs. LHS groups. (**A**) Volcano plot of DAMs in the HHS vs. LHS comparison. (**B**) OPLS-DA score plot demonstrating intergroup separation. (**C**) Hierarchical clustering heatmap of scaled relative abundances of DAMs. (**D**) Correlation network of representative DAMs. The *y*-axis and the diagonal *y*-axis both represent the names of the DAMs. The colors indicate the correlations, with cyan representing positive correlation and brown representing negative correlation. The deeper the color, the stronger the correlation. A blank indicates no significant correlation (*p*-value > 0.05). (**E**) KEGG enrichment analysis of DAMs.

LC-MS/MS analysis was conducted to investigate the variations in metabolite composition associated with the meat diversity in sheep with different carcass performances. We detected a total of 938 metabolites in the HHS vs. LHS comparison. Among these, 132 were classified as DAMs, with 20 up-regulated and 112 down-regulated in the HHS group (Figure 5A; Table S4). OPLS-DA and hierarchical clustering heatmap analysis of scaled relative abundances of differentially accumulated metabolites demonstrated distinct metabolic patterns between the two groups (Figure 5B,C). Pearson correlation analysis of all metabolites was performed using the stats package (v4.4.1) in the R environment (v4.4.1), with a *p*-value < 0.05 indicating a significant correlation. Correlation analysis of the DAMs revealed both positive and negative correlations. For instance, “arachidoyl ethanolamide” and “(2R)-3-(hexadecyloxy)-2-[(5Z,8Z,11Z,14Z)-5,8” exhibited a positive correlation (cor = 0.71), whereas “2-bromo-6-chlorobenzoic acid” and “flavin adenine dinucleotide (FAD)” showed a negative correlation (cor = −0.78, Figure 5D). The KEGG pathway enrichment analysis showed that most of the DAMs were involved in metabolism-related pathways, including “biosynthesis of amino acids”; “D-amino acid metabolism”; “arginine biosynthesis”; “valine, leucine, and isoleucine biosynthesis”; and “glycine, serine, and threonine metabolism” (Figure 5E; Table S5).

### 3.6. Integrative Analysis of the Transcriptome and Metabolome

**Figure 6:**
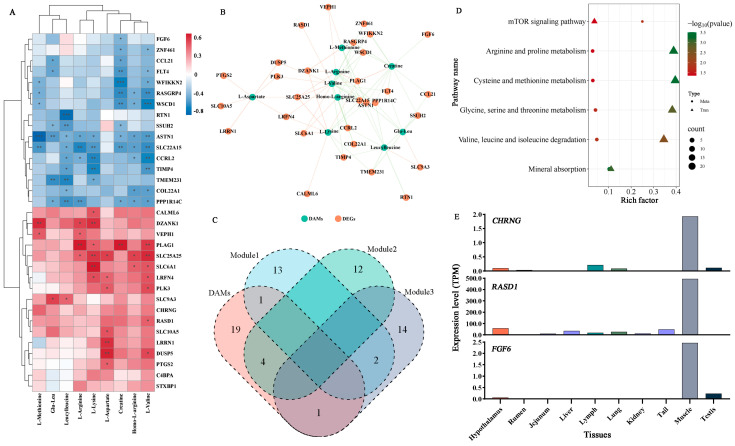
Integrative analysis of transcriptomic and metabolomic networks. (**A**) Pearson’s correlations between DEGs and DAMs in the HHS vs. LHS groups. * *p*-value < 0.05; ** *p*-value < 0.01; *** *p*-value < 0.001. (**B**) Correlation network between DEGs and DAMs in the HHS vs. LHS groups. Red lines represent positive correlations, while green lines represent negative correlations. The thickness of the lines indicates the strength of the correlation coefficient. (**C**,**D**) Common KEGG pathways between WGCNA modules and DAMs. (**E**) Tissue-specific expression patterns of key genes in muscle tissues.

Based on the correlation strength, we present the top 20 gene-metabolite pairs that exhibited significant positive or negative correlations. Figure 6A,B show the genes with positive and negative correlations (|r| > 0.5 and *p* < 0.05) to nine metabolites associated with meat quality. For instance, *PLAG1*, *CHRNG*, *SLC9A3*, *LRRN1*, and *DUSP5* are involved in metabolic processes as well as the development of tissues and organs in animals and have been associated with amino acids affecting meat quality. Subsequently, we performed a combined analysis of the enrichment pathways for the three gene modules identified through WGCNA and the pathways enriched in DAMs (Figure 6C). The results revealed six co-enriched pathways, including “mineral absorption”; “valine, leucine, and isoleucine degradation”; “mTOR signaling pathway”; “glycine, serine, and threonine metabolism”; “arginine and proline metabolism”; and “cysteine and methionine metabolism” (Figure 6D). Additionally, our analysis of previous research data [12] revealed that several genes are highly expressed in sheep muscle tissue, including *CHRNG*, *RASD1*, and *FGF6* (Figure 6E).

### 3.7. qPCR Analysis

**Figure 7:**
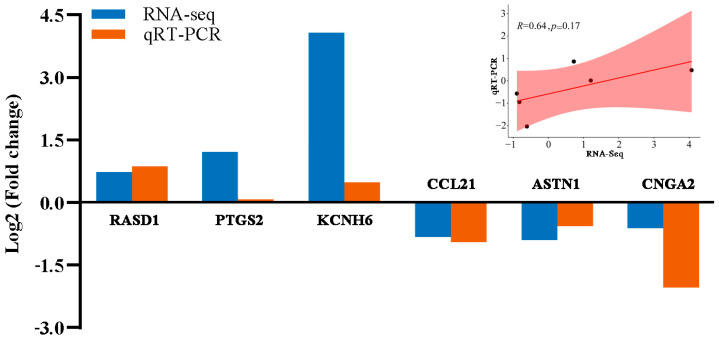
qRT-PCR validation of RNA-Seq data.

To confirm the RNA-Seq data, we employed qPCR to measure the mRNA expression levels of DEGs. As shown in Figure 7, qPCR results for the tested genes showed partial agreement with the RNA-Seq data regarding the direction of change for most genes, although the magnitude of fold changes differed for some. While these qPCR results provide some support for the RNA-Seq findings, the limited number of genes validated and the lack of statistically significant correlation highlight the need for future validation with a larger sample size.

## 4. Discussion

The priorities of stakeholders in the meat supply chain often diverge, with processors and retailers emphasizing carcass quality and lean meat yield, while consumers place greater emphasis on eating quality and nutrition [4]. Meat quality traits are key economic factors in livestock production. In recent years, mutton production in China has steadily risen; however, the demand for high-quality mutton remains unmet [29]. The meat quality of lamb is shaped by genetic, dietary intake, age, and gender-related aspects [30]. Genetic improvement can be achieved through direct selection for quantitative traits or by using marker-assisted and genomic selection [31]. This research examined the variations in meat quality among Hu sheep with varying carcass performance and identified the key molecular markers responsible for these variations. The correlation analysis results indicate a non-significant, weak positive correlation trend between muscle fiber indicators and shear force, which is consistent with previous studies that show a negative correlation between muscle fiber diameter and meat tenderness [32]. As expected, the high-carcass-performance group exhibited higher backfat thickness, GR value, intramuscular fat, marbling score, and muscle weight compared to the low-carcass-performance group. Conversely, the high-carcass-performance group demonstrated lower shear force, drip loss, and cooking loss than the low-carcass-performance group. Higher intramuscular fat content improves muscle tenderness and flavor [33]. The marbling score, a visual indicator of intramuscular fat, significantly impacts meat quality by positively influencing juiciness, flavor preference, and overall sensory appeal [34,35]. Shear force and water-holding capacity are key indicators of meat tenderness and juiciness, which significantly influence consumer acceptance [36,37]. Thus, our data suggest that muscle tenderness in high-carcass-performance sheep may be superior to that in low-carcass-performance sheep.

The identification of 376 DEGs between high- and low-carcass-performance sheep underscores the genetic complexity influencing meat quality. The enrichment of DEGs in pathways such as “brown fat cell differentiation”, “positive regulation of developmental process”, and “peroxidase activity” supports earlier research highlighting the importance of lipid metabolism in regulating IMF content and marbling, which are crucial factors in meat quality [5,38]. Up-regulated of *ELOVL6* and *PLIN1* in HHS sheep—genes implicated in fatty acid elongation and lipid droplet stabilization [39,40]—suggests enhanced lipid synthesis and storage capacity. The *PTGS2* gene is differentially expressed in the muscle of Tianzhu white yaks at various developmental stages and contributes to muscle growth and fat accumulation [17]. The *SLC22A4* gene may be associated with fat-tail dimensions in sheep, indicating its potential involvement in the regulation of fat deposition [41]. Shao et al. [27] demonstrated an association between the *ADIPOQ* gene and intramuscular fat content in the longest dorsal muscle of sheep. These findings corroborate the importance of lipid metabolic networks in shaping IMF content, a key determinant of meat juiciness and flavor. The WGCNA further identified co-expression modules (MEblue, MEyellowgreen, and MEsalmon) strongly associated with cooking percentage, cooking loss, and shear force. The enrichment of oxidative phosphorylation, fatty acid biosynthesis, and amino acid metabolism pathways within these modules implies that mitochondrial energy metabolism and fat content influence water-holding capacity and tenderness. For instance, *ARHGEF2*, a hub gene in the MEyellowgreen module, is linked to intramuscular fatty acid composition in porcine [42], which plays a crucial role in meat tenderness. The *SLC43A2* gene has been reported to be up-regulated in sheep with high unsaturated fatty acid content, suggesting a potential association with meat quality and flavor [43]. It has been suggested that body fat, as measured by BMI, may play a role in regulating the expression of the adipose *ACACB* gene in humans, which could have implications for metabolic processes and adiposity-related traits [44]. *SEMA4C* has been shown to be essential for the development of skeletal muscle in zebrafish, indicating its potential role in muscle formation and growth [45]. While these findings provide valuable preliminary insights into potential gene functions, it is important to acknowledge that evidence derived from distinct species such as yaks, humans, zebrafish, and pigs may not fully translate to sheep biology. Consequently, functional validation in sheep is essential to substantiate these proposed roles.

Metabolomic analysis identified 132 DAMs between the HHS and LHS groups, with significant enrichment in amino acid metabolism pathways. The metabolites related to the biosynthesis of amino acids like valine, leucine, and isoleucine suggest a potential link between amino acid metabolism and meat quality. These metabolites are essential for protein synthesis and energy production, and their altered levels may impact muscle development and postmortem biochemical processes [46]. The observed correlations between specific metabolites and meat quality traits provide valuable insights into the metabolic reprogramming associated with carcass performance. The integrative analysis of transcriptomic and metabolomic data revealed six co-enriched pathways, including mineral absorption; valine, leucine, and isoleucine degradation; and the mTOR signaling pathway. These pathways are integral to nutrient metabolism and muscle development, further emphasizing their role in regulating meat quality [47,48]. The identification of key genes such as *CHRNG*, *RASD1*, and *FGF6*, which are highly expressed in sheep muscle tissue, highlights the unique genetic factors contributing to meat quality in Hu sheep. The observed correlations between specific genes and metabolites suggest potential associations within molecular networks that may underlie variation in meat quality traits. Our findings identify potential candidate biomarkers for genetic selection and nutritional strategies aimed at enhancing both carcass yield and meat quality in sheep, pending future functional validation. The identification of key DEGs and DAMs associated with desirable meat quality traits provides a foundation for marker-assisted selection in breeding programs. Additionally, the insights into metabolic pathways and regulatory networks offer potential targets for nutritional interventions aimed at optimizing muscle development and fat deposition. By integrating these molecular insights into breeding and nutritional strategies, it is possible to enhance the economic value and consumer preference for sheep meat.

## 5. Conclusions

Overall, this research provides new insights into the molecular mechanisms underlying meat quality variation in Hu sheep with different carcass performance. Our findings demonstrate that the high-carcass-performance group exhibited superior meat quality traits, characterized by significantly higher IMF content and marbling score, alongside lower shear force, drip loss, and cooking loss. Through integrated transcriptomic and metabolomic analyses, we identified key DEGs and DAMs associated with meat quality traits. Functional enrichment analyses of these DEGs and DAMs revealed that pathways central to lipid metabolism, amino acid metabolism, and energy utilization play crucial roles in regulating meat quality in Hu sheep. Several hub genes such as *ELOVL6*, *PLIN1*, *ARHGEF2*, *PLAG1*, and *CHRNG*, along with metabolites involved in amino acid biosynthesis and degradation, were strongly correlated with desirable meat quality traits. This integrated approach allowed us to delineate the complex genetic and metabolic networks underpinning meat quality traits. Collectively, our work provides new mechanistic insights into the basis of meat quality variation and identifies concrete potential biomarkers that could inform future genetic selection programs and targeted nutritional strategies to enhance meat quality in Hu sheep production.

## Figures and Tables

**Table 1:** Parameters of primer pairs used for qRT-PCR.

Gene Name	Primer Sequences (5′–3′)	Annealing Temperature (°C)	Product Length (bp)
*RASD1*	GAGGACTTCCACCGCAAGTTCTAC	60.5 °C	144 bp
	CAGGCTGAACACCAGGATGAACAC		
*PTGS2*	CCAGACAAGTAGGCTAATCCTG	53.3 °C	160 bp
	GTTAAACTCAGCAGCAATACGG		
*KCNH6*	ACACCATCATCCGCAAGTTCG	56.3 °C	106 bp
	ACACCATCATCCGCAAGTTCG		
*CCL21*	TCACTGGTCCTGAGCATCCTTGT	61.2 °C	124 bp
	CAATGTTGGCGGGAATCTTCTTTCG		
*ASTN1*	ATCTCAGGCAACACGGAGGACAT	60.3 °C	141 bp
	ATGCTGTGGTTCTTCGGTTGGATC		
*CNGA2*	CCCAACATCTTCCGAATCAGC	54.6 °C	105 bp
	TACCCCAAAGCCGATGGAC		
*UXT*	GCAAGTGGATTTGGGCTGTAAC	58.5 °C	180 bp
	ATGGAGTCCTTGGTGAGGCTGT		

**Table 2:** Growth and slaughter traits of 20 Hu sheep.

	HHS	LHS	*p*-Value
No.	10	10	
Body weight (kg)	50.26 ± 1.01	37.44 ± 0.90	<0.001
Body length (cm)	69.30 ± 1.08	64.70 ± 0.79	0.003
Body height (cm)	77.00 ± 0.71	71.30 ± 0.86	<0.001
Chest circumference (cm)	91.80 ± 1.35	83.40 ± 1.17	<0.001
Body mass index (kg/cm^2^)	0.0085 ± 0.00021	0.0074 ± 0.00015	<0.001
Live weight before slaughter (kg)	51.27 ± 0.89	37.27 ± 0.63	<0.001
Carcass weight (kg)	28.06 ± 0.31	19.96 ± 0.11	<0.001
Carcass length (cm)	86.50 ± 1.15	79.80 ± 0.61	<0.001
Chest circumference of carcass (cm)	78.50 ± 0.81	70.70 ± 0.45	<0.001

Notes: Phenotypic values are presented as the mean ± SE, with *p*-value calculated using a *t*-test.

## Data Availability

The RNA-Seq data of 20 Hu sheep from the study were deposited in the NCBI Sequence Read Archive (SRA) under BioProject accession number PRJNA1289230. Metabolomics data will be made available on request.

## Supplementary Materials

The following supporting information can be downloaded at https://www.mdpi.com/article/10.3390/foods14142477/s1. Figure S1: (A) Soft-threshold power selection: (Left) Scale independence analysis showing optimal power at 8; (B) cluster analysis of the relationships between different modules; (C) module gene correlation heat map. Table S1: List of differentially expressed genes in the HHS vs. LHS comparison. Table S2: GO term enrichment analysis of DEGs. Table S3: KEGG pathway enrichment analysis of DEGs. Table S4: List of differentially accumulated metabolites in the HHS vs. LHS comparison. Table S5: KEGG pathway enrichment analysis of 132 DAMs.

## Abbreviations

The following abbreviations are used in this manuscript:

DEGs	Differentially expressed genes
DAMs	Differentially accumulated metabolites
WGCNA	Weighted gene co-expression network analysis
TOM	Topological overlap matrix
LT	Longissimus thoracis
TPM	Transcripts per million
GO	Gene ontology
KEGG	Kyoto Encyclopedia of Genes and Genomes
IMF	Intramuscular fat
