# Supplementary figures and images for: Integrative Analysis of Transcriptomics and Metabolomics Provides Insights into Meat Quality Differences in Hu Sheep with Different Carcass Performance
Source: Foods. 2025 Jul 15;14(14):2477. doi: 10.3390/foods14142477 (PMC12294501; doi:10.3390/foods14142477)

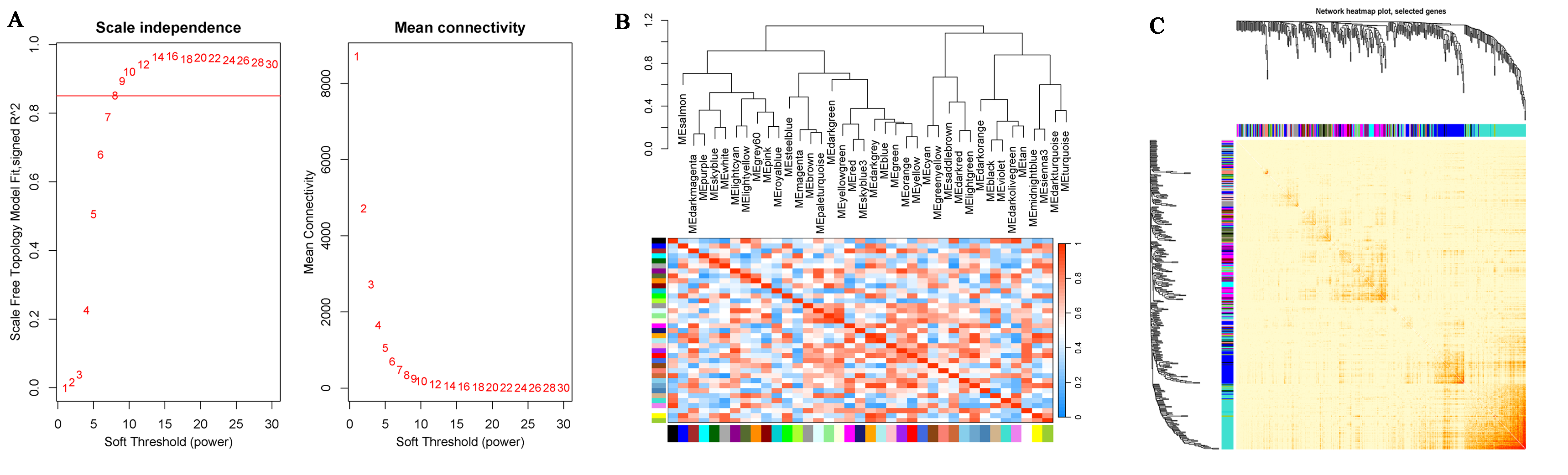

Supplement: Supplementary file 1 [file foods-14-02477-s001.zip › Figure S1.tif]
